# A facility-based study of women’ satisfaction and perceived quality of reproductive and maternal health services in the Kenya output-based approach voucher program

**DOI:** 10.1186/s12884-018-1940-9

**Published:** 2018-07-28

**Authors:** Boniface Oyugi, Urbanus Kioko, Stephen Mbugua Kaboro, Clarice Okumu, Sarah Ogola-Munene, Shaminder Kalsi, Simon Thiani, Shadrack Gikonyo, Julius Korir, Billy Baltazar, Moses Ranji

**Affiliations:** 1 University of Nairobi Enterprise and Services Consultancy, Arboretum Drive, P.O BOX 68241-00200, Nairobi, Kenya; 2 School of Public Health, Health Systems Management, University of Nairobi, P.O BOX 19676-00202, Nairobi, Kenya; 3 Centre for Health Services Studies, University of Kent, Canterbury, CT2 7NX UK; 4 OBA Program Management Unit, Ministry of Health, Nairobi, Kenya

**Keywords:** Quality perception, Satisfaction, Output based approach, Vouchers, Reproductive health

## Abstract

**Background:**

This is a facility-based study designed to assess perceived quality of care and satisfaction of reproductive health services under the output-based approach (OBA) services in Kenya from clients’ perspective.

**Method:**

An exit interview was conducted on 254 clients in public health facilities, non-governmental organizations, faith-based organizations and private facilities in Kitui, Kilifi, Kiambu, and Kisumu counties as well as in the Korogocho and Viwandani slums in Nairobi, Kenya using a 23-item scale questionnaire on quality of reproductive health services. Descriptive analysis, exploratory factor analysis, reliability test, and subgroup analysis using linear regression were performed.

**Results:**

Clients generally had a positive view on staff conduct and healthcare delivery but were neutral on hospital physical facilities, resources, and access to healthcare services. There was a high overall level of satisfaction among the clients with quick service, good handling of complications, and clean hospital stated as some of the reasons that enhanced satisfaction. The County of residence was shown to impact the perception of quality greatly with other social demographic characteristics showing low impact.

**Conclusion:**

Majority of the women perceived the quality of OBA services to be high and were happy with the way healthcare providers were handling birth related complications. The conduct and practice of healthcare workers is an important determinant of client’s perception of quality of reproductive and maternal health services. Findings can be used by health care managers as a guide to evaluate different areas of healthcare delivery and to improve resources and physical facilities that are crucial in elevating clients’ level of satisfaction.

**Electronic supplementary material:**

The online version of this article (10.1186/s12884-018-1940-9) contains supplementary material, which is available to authorized users.

## Background

Improving maternal and child health are critical priorities in enhancing the agenda of quality of healthcare to some of the most vulnerable groups [1–4]. Despite substantial progress and different strategies that have been implemented by different countries, decline in maternal and child mortality remains inadequate [5–7]. Maternal and child mortality is largely preventable with current technology and it is unjustly and inequitably borne by low and middle income countries with poorly resourced health systems [8]. Findings from the Kenya Demographic Health Survey (2014) confirm that more effort is still needed towards reducing child mortality and improving maternal health despite the progress that has been made [9].

The quality of healthcare services plays an important role in enhancing healthcare service delivery in low income countries [10]. Poor quality of healthcare may lead to under-utilization of services; and evidence shows that pregnant women are more likely to deliver in health facilities if they are content with the care that they receive at the service delivery points [11, 12]. A study conducted in rural Zimbabwe found that poor quality of services and negative attitudes of health care workers hinder pregnant women from utilizing these services [13]. Where poor women have access to what they perceive as high quality health care services, they increasingly seek reproductive health care services and delivery in health facilities [14].

### Overview of the output based approach reproductive health program

Evidence from various studies has shown that there are significant direct and indirect cost barriers in seeking reproductive and maternal health services, including treatment of complications [8]. Furthermore, high expenditures arising from birth related complications hinder many poor mothers from accessing health care and may push households further into poverty [15].

Two governments, Kenya and Germany, came together in 2005 to jointly support reproductive health through the Output Based Approach (OBA) Program. The purpose of the program is to expand utilization of selected reproductive health services among women aged 15–49 years (reproductive age). The program targets mothers who are economically disadvantaged and living in the counties of Kisumu, Kitui, Kiambu, and Kilifi, in addition to those who are in Korogocho and Viwandani, which are informal settlements in the county of Nairobi. The reproductive services offered include: safe motherhood (SMH) which comprise antenatal care (ANC) attendance, caesarian section and vaginal delivery, birth related complication and post-natal care up-to 6 weeks after delivery. Additionally, the program supports long-term family planning (LTFP) methods such as intra-uterine contraceptive device (IUCD), implants, and tubal ligation. Equally, the program offers counselling, medical examination, and treatment to vulnerable mothers who encounter sexual gender-based violence as has been shown by other authors [16, 17].

OBA aims to support the impoverished population through subsidized health services [17]. The program pays service providers on the basis of agreed outputs with pre-defined results, e.g. facility-based deliveries and antenatal care visits attended, rather than financing the inputs [15]. Under the OBA model, vouchers for safe motherhood (SMH) and long-term family planning (LTFP) services are sold at highly subsidized prices to prospective women (100 Kenya shillings for both Family planning and the safe motherhood in Kilifi County and 200 Kenya Shillings for safe motherhood and 100 Kenya Shillings for family planning in other counties – 1 USD ($) is approximately 100 Kenya shillings). For each voucher presented to accredited health facilities (including private providers, government facilities, non-governmental organizations - NGOs, and faith-based organizations - FBOs), services are provided and facilities reimbursed at a fixed rate [8, 15, 16, 18, 19]. Facilities are expected to use the reimbursed funds to improve infrastructure, purchase some medical and non-medical supplies, and provide incentives to facility staff among other things. The program directly finances the beneficiaries with highly subsidized vouchers, and funding is reimbursed directly to accredited health facilities.

Donabedian theory evaluates three categories of quality of care: structure, which include inputs such as equipment and personnel, process which focusses on the activities carried out by the personnel, and outcomes which focuses on improved patient health such as good recovery, survival, and client satisfaction [20–22]. While the program has been in existence since 2005, little research has been done on aspects of patient perception of quality of reproductive healthcare. For instance, one study on quality of the safe motherhood voucher schemes showed enhanced quality of post birth care and a likelihood of superior quality of care for clients who opted to participate in the voucher scheme for longer [23]. The study evaluated only the postnatal aspect of care and did not touch on quality issues in overall totality. Hence, there is a paucity of data on quality of reproductive care, satisfaction with OBA services, and the impact of such programs. Therefore, this study evaluated perceived quality and satisfaction of the services under the OBA voucher program in Kenya from a woman’s perspective. Additionally, we evaluated predictors of the factors that are related to perceived quality of reproductive care in OBA facilities.

## Methods

### Study area

The study was conducted in Kitui, Kilifi, Kiambu, and Kisumu counties as well as in the Korogocho and Viwandani slums in Nairobi which are the OBA program sites. The services in OBA sites are provided by public, NGOs, FBOs, and private service providers. All participating sites were offering SMH services (ANC, Delivery, treatment of delivery complications, and post-natal care up to 2 weeks), LTFP methods, and a small number was providing SGBV services.

### Study design and tool

This was a cross-sectional study conducted in OBA sites using a semi-structured interview guide administered through face-to-face in-depth exit interviews. Participants receiving OBA services were asked to describe their perceptions of the quality of services and reasons for satisfaction with the quality of services they had received in their current and previous visits. Perception was measured using a questionaire (Additional file 1) that was developed on the basis of literature review and suited for a healthcare setting [10, 24]. The questionaire consisted of a large number of items that were found to be imperative in measuring quality of and satisfaction with care. Women were specifically asked how they perceived the care they received during SMH visits, LTFP visits, and SGBV visits. Besides, they were also asked about the information they received, the conduct of the healthcare professionals, and adequacy of resources and services. The items were re-grouped into 23 items measuring perception. There were two additional questions; one, on whether the women were completely satisfied with the services and two, on the reasons for satisfaction or dissatisfaction. Perceived quality of services was rated on a five point Likert Scale 1 being “Completely Disagree”, 2 “Disagree”, 3 “Agree”, 4 “Completely Agree”, and 5 “Do Not Know”.

### Sampling design

In selecting participants, a multistage sampling technique was used to select the facilities offering OBA services. First, all OBA facilities were classified according to type of ownership-public and private and grouped according to County. Classification has been described elsewhere [16]. Within each County, a representative sample of facilities both public, NGOs, FBOs and private facilities was randomly selected. In the second stage, a conservative sample size was calculated to be 313 respondents. In order to determine the sample size the formula developed by Cochran [25] for proportion that are larger: *n* = z^2^pq/d^2^, where *n* = was the number of clients/respondents, z = is the critical value for standard normal distribution for the 95% confidence interval around the true population (1.96), *p* = estimated proportion utilising OBA services (which was based on the proportion of women of reproductive age living below the poverty line in Kitui, Kiambu, Nairobi, Kisumu and Kilifi estimated at 28.56% [26]), q = represented 100-p, and d = was the degree of accuracy (5%). The number of clients were equally divided amongst the chosen facilities (5 clients). A simple random technique was used to select the OBA clients who sought SMH, LTFP, and SGBV care at the time of the study. To randomly select the participants at the facility, the researchers used Stat Trek Random numbers generators which have been applied in other cross sectional studies [27]. The method uses statistical algorithm to give random numbers and instructions on how to use it (http://stattrek.com/statistics/random-number-generator.aspx). The researchers hit a calculate button and the number generator gave a random number table with five numbers between 1 and 20. Subsequently, the interviewers then interviewed the participants presented by these numbers on a single basis until the sample size was obtained. After data collection, the questionaires were then retured to the central OBA program management offices in Nairobi after which they were checked for completeness before inclusion into the database. Only fully completed questionaires with all essential details were included in the analysis and “do not know” response in the questionaire was treated as a neutral term for ease of interpretation.

### Data analysis

The data were analysed using Statistical Packages for Social Scientists (SPSS) version 18. Descriptive statistical analysis was carried out to describe the respondents’ social demographic characteristics and the time taken to reach the facility either by bus or by foot. Additionally, descriptive statistical analysis was conducted on the women’ perceptions of OBA services. Data were then subjected to exploratory factor analysis (EFA) of the 23 items to break down the items into homogonous sub-scales coherent with the quality dimentions as proposed by Donabedian [20]. Principal component analysis with orthogonal varimax rotation was conducted. In addition, the Kaiser-Meyer-Olkin measure (KMO) was done to evaluate the suffiency of data for EFA and Bartlett’s test of sphericity to evaluate the degree of patterned relationship between the items. Additionaly, reliability analysis was performed to test the reliability of the scale and internal consistencies of extracted factors; whereby Cronbach’s alpha coefficient was calculated. The multivariate response model was used to study whether level of education, ante-natal clinic visit, marital status, age, and County of residence were predictors of the factors related to perceived quality of reproductive care (Table 1). The questions on overal satisfaction and reasons for satisfaction were analysed using Microsoft excel 2010 and Pareto chart [28] was obtained for the level of satisfaction.

**Table 1:** Definition and measurement of variables used in multi linear regression model

Variable definition	Measurement
Outcome variable	
Factors related to perceived quality of reproductive care	Staff conduct and practice, Healthcare delivery, Physical facilities, adequacy of resources, Accessibility of care, Perceived quality (Total Score)
Independent variables	
Level of education	1 = No education, 2 = Primary level, 3 = Secondary level, 4 = Tertiary level
Attendance to ANC clinic	1 = Two or less, 2 = Three times or more
Marital Status	1 = Never Married, 2 = Married, 3 = Separated/Divorced, 4 = Other
Age	1 = 24 and below, 2 = 25–34 years, 3 = 35–44 years, 4 = 45 years and above
County of residence	1 = Nairobi, 2 = Kiambu, 3 = Kilifi, 4 = Kisumu, 5 = Kitui

### Ethical approval

The authorization to carry out the study was obtained from the Ministry of Health-Kenya as part of routine monitoring of the process (Development of the Health Sector, Health Financing Support and Output Based Approach, Phase III, BMZ-No. KENYA 2010 65853) of the OBA services. The proposal was approved by the health research unit of the Ministry of Health Kenya (MOH/HRD/1/ (32)). Additionally, permission was obtained from the county headquarters and hospital administrators to proceed with the study. Verbal informed consent for the study was obtained from every woman who agreed to participate. The interviewers explained the purpose of the study to the mothers in their local dialect (language) and asked them whether they were willing to participate. For those who agreed, the interviewer indicated a unique patient identifier and the date of the interview on the front page of the questionnaire before proceeding with the interview and data were only used for the study.

## Results

The study was conducted in 65 OBA accredited facilities (18 FBOs, 2 NGOs, 18 private, and 27 public) in Kiambu, Nairobi, Kilifi, Kisumu, and Kitui (Table 2).

**Table 2:** Number of facilities where interviews were conducted

	FBO	NGO	Private	Public	totals
Kiambu	3	0	3	3	9
Nairobi	1	1	1	1	4
Kilifi	2	0	3	8	13
Kisumu	5	1	5	6	17
Kitui	7	0	6	9	22
Total	18	2	18	27	65

### Socio-demographic data of the respondents

Out of a sample of 313 respondents, 254 were included for analysis making the response rate 81.2%. Fifty nine questionnaires that had no imperative details on the independent variables (levels of education, attendance to ANC clinic, marital status, and age) and where more than two attributes of quality were missing, were excluded from the analysis. The details were considered important to avoid bias in the multivariate response model and exploratory factor analysis as was shown in other studies [10, 29]. There were 198 women with Safe Motherhood (SMH) contacts, 55 with Long Term Family Planning (LTFP) contacts, and one with a Sexually Gender Based Violence (SGBV) contact. All respondents were female, most of them married (83.1%) with primary level of education (57.9%). Majority of the respondents were in the age group of 24 and below (53.9%) followed by those in the age group 25–34 years old (38.6%) (Table 3) below. Mean age of the respondents was 24.67 years old (SD 6.127), and mean time taken to get to the facilities by foot and bus was 93.95 min (SD 304.877) and 36.83 min (SD 43.993) respectively. Additionally, majority of the women had attended ANC clinics “three times or more” (76%).

**Table 3:** Socio demographic characteristics of the respondents

Variable			Frequency (%)
Age	24 and below		137(53.9)
	25–34		98(38.6)
	35–44		19(7.5)
	45+		0(0)
Marital status	Single		32(12.6)
	Married		211(83.1)
	Separated/ Divorced		11(4.3)
	Other		0(0)
Number of ANC visits attended	Two or less		61 (24)
	Three times or more		193 (76)
Education	No Education		16(6.3)
	Primary		147(57.9)
	Secondary		68(26.8)
	Tertiary		23 (9.1)
	Age	Time by Foot in minutes	Time by vehicle in minutes
Mean	24.67	93.95	36.83
Median	24	30	30
Std. Deviation	6.127	304.877	43.993
Range	43	4320	300

### Women’ perception of services provided

The overall mean score for women’ perception of quality of services was 3.43 (SD 0.629) (Table 4), implying that the majority perceived the quality of OBA services to be high. Specifically, women were happy with the way healthcare providers were handling birth related complications. Furthermore, women highly rated staff as “compassionate”, “respectful”, “able to prescribe drugs that are needed”, and “able to examine post-partum women well.” However, the adequacy of the number of facility staff was rated fairly low implying that some facilities did not have enough staff.

**Table 4:** Mean scores of perceived quality of care in the OBA sites

Attributes	Mean	SD
Number of Staff in the facility is adequate	3.10	0.826
Waiting rooms, examination rooms, and other rooms are adequate	3.24	0.757
Clean water for clients is adequate	3.31	0.777
Hand washing facilities for clients is adequate	3.33	0.691
Bathing facilities for clients is adequate	3.42	0.878
Toilet facilities for clients is adequate	3.28	0.785
Environment of the facility is clean	3.35	0.629
Equipment is well suited for detecting medical problem	3.46	0.722
Complications handled satisfactorily	3.62	0.623
There is enough privacy while handling cases	3.32	0.653
You received enough information for the services to help you make decisions	3.44	0.586
Staff examine post-partum women well	3.55	0.607
Staff prescribe drugs that are needed	3.55	0.566
There is adequate supply of drugs in facility	3.37	0.688
Patient can easily obtain drugs from the facility	3.43	1.345
Information provided on danger sign adequate	3.30	0.705
Staff have adequate knowledge of dealing with FP, Deliveries, CS, SGBV cases	3.54	0.581
Staff very capable of finding what is wrong with patients	3.53	0.553
Staff are OPEN	3.51	0.595
Staff are Compassionate	3.56	0.557
Staff are Respectful	3.56	0.586
Staff have adequate devotion to clients	3.51	0.575
Staff are Honest	3.54	0.627
Overall mean for the 23 attributes	3.43	0.629

### Factor analysis results

Principal component analysis with orthogonal varimax rotation was conducted where the Kaiser-Meyer-Olkin measure (KMO) was 0.893 well above 0.5 suggested by Kaiser, 1974 [30] as shown in Table 5 indicating that the data was sufficient for exploratory factor analysis (EFA). The Bartlett’s test of sphericity X2 (276) = 2866.439, *P* < 0.001 (Table 5) showed that there was some degree of patterned relationship between the items. Items that had measures of variance (eigenvalues) equal to or greater than 1, with factor loading above 0.4, and factors that had three or more items were retained and used for EFA [29]. EFA used five factors which accounted for 61.5% of variance explained by the data after extraction. These were used in defining five sub-scales (Table 5). All five factors were included in the analysis because each had more than three variables as suggested by Hair et al. [29]. The five factors were labeled as follows: F1- “Staff conduct and practice” which had five variables (Staff are compassionate, staff are respectful, staff are devoted to clients, staff are open, staff are honest) and explained most of the variance; F2- “Healthcare delivery” which had seven variables (Staff very capable of diagnosing patient’s illness, complications handled satisfactorily, staff examined post-partum women well, client received adequate information for the services to help making informed decisions, equipment is well suited for detecting medical problems, staff prescribed drugs that are needed, and staff have adequate knowledge in dealing with family planning issues, vaginal deliveries, caesarean deliveries, sexual and gender based violence cases); F3- “physical facilities” which had five variables (Clean water is adequate, there is enough privacy while handling cases, toilet facilities are adequate, hand washing facilities are adequate, environment of the facility is clean); F4- “adequacy of resources” which had three variables (Information provided on danger signs is adequate, bathing facilities for clients is adequate, number of Staff in the facility is adequate); and F5- “Accessibility of care” which had three variables (Patient can easily obtain drugs from the facility, there is adequate supply of drugs in facility, waiting rooms, examination rooms, and other rooms are adequate). Most of the factor loading were greater than 0.4 and the communalities ranged from 0.815 to 0.499 showing that the factor solution had identified the variance associated with each factor.

**Table 5:** Factor analysis result of OBA clients’ perceptions (n=254)

Rotated Component Matrix						
	Factors					Communalities after extraction
	F1	F2	F3	F4	F5	
Staff are Compassionate	**0.865**					0.815
Staff are Respectful	**0.861**					0.808
Staff have adequate devotion to clients	**0.790**					0.723
Staff are OPEN	**0.756**					0.668
Staff are Honest	**0.668**					0.559
Staff have adequate knowledge of dealing with FP, Deliveries, CS, SGBV cases		**0.753**				0.679
Staff very capable of finding what is wrong with patients		**0.691**				0.688
Complications handled satisfactorily		**0.656**				0.537
Staff examine post-partum women well		**0.619**				0.531
You received enough information for the services to help you make decisions		**0.558**				0.605
Equipment is well suited for detecting medical problem		**0.540**		0.469		0.528
Staff prescribe drugs that are needed		**0.501**				0.528
Clean water for clients is adequate			**0.746**			0.589
There is enough privacy while handling cases			**0.656**			0.663
Toilet facilities for clients is adequate			**0.635**	0.402		0.643
Hand washing facilities for clients is adequate			**0.613**			0.548
Environment of the facility is clean	0.438		**0.610**			0.699
Information provided on danger sign adequate				**0.696**		0.524
Bathing facilities for clients is adequate				**0.580**		0.510
Number of Staff in the facility is adequate		0.444		**0.573**		0.567
Patient can easily obtain drugs from the facility					**0.799**	0.655
There is adequate supply of drugs in facility					**0.469**	0.570
Waiting rooms, examination rooms, and other rooms are adequate					**0.447**	0.499
Eigenvalue	8.502	1.929	1.507	1.121	1.077	14.136
% of variance explained by the factor after extraction	18.12	14.94	12.34	9.42	6.64	61.46
Extraction Method: Principal Component Analysis; Rotation Method: Varimax with Kaiser Normalization.						
Kaiser-Meyer-Olkin Measure of Sampling Adequacy						0.893
Bartlett's Test of Sphericity	Approx. Chi-Square					2866.439
	Df					276
	Sig.					.000

Bold shows items that converge to form a factor

### Reliability analysis results

The reliability (internal consistency) of the sub-scales exhibited by Cronbach’s alpha ranged from 0.525 for F5 (showing low internal consistence) to 0.904 for the total score (indicating high internal consistence) (Additional file 2: Table S1 shows this in more details). The slightly lower scores for F4 and F5 can be explained by the small number of items in the group and has been explained by writers such as Haddad et al. [24]. Means of all five factors were fairly above three and they were fairly equal to median scores showing that there was no skewed distribution on the perception of the women.

### Socio-demographic predictors of quality of reproductive health services

Regression analyses were performed with the different sub-scales and the total score for perceived quality of OBA services as outcome variables. The B values (beta) were interpreted directly as shown in Additional file 3: Table S2 and Additional file 4: Table S3. The results of the regression analyses indicate that marital status and the number of Ante Natal clinic (ANC) visits play insignificant roles in determining the perception of quality of OBA services within different factors except for the overall perceived quality of reproductive health care (Additional file 3: Table S2 and Additional file 4: Table S3). However, counties (areas of residence) are a significant determinant of the level of perception of quality. For instance, four factors (staff conduct and practice, physical facilities, adequacy of resources, accessibility of care) and the total score are perceived poorly by women in Nairobi, Kitui, Kilifi, and Kisumu as compared to Kiambu County (reference category).

The results showed that staff conduct and practice is perceived poorly by those aged 15–25 years as compared to those aged 25–34; and perceived poorly by those with primary education as compared to those with secondary education. Healthcare delivery is judged poorly by those with tertiary education as compared to women with primary education, and poorly by those aged 15–24 compared to those aged 25–34 years old. Additionally, physical facilities are perceived positively by those without education or with secondary education as compared to primary education. Those without education perceive adequacy of resources more favorably than those with primary education. Accessibility of care is judged negatively by individuals aged 15–24 and 34–44 years as compared to individuals aged 25–34 years old. Overall, the quality of OBA services was judged higher by both those without education and with secondary education compared to those with primary education, and those who have attended two or less ANC visits compared to those who attended three times or more.

The variance explained by various factors (R^2^) is higher than 10% for staff conduct and practice, healthcare delivery, physical facilities, and adequacy of resources. In general, this shows that only for perceived staff conduct and practice and for perceived adequacy of resources, a substantial part of the variance is explained by socio-demographic factors.

### Overall level of satisfaction

**Fig. 1:**
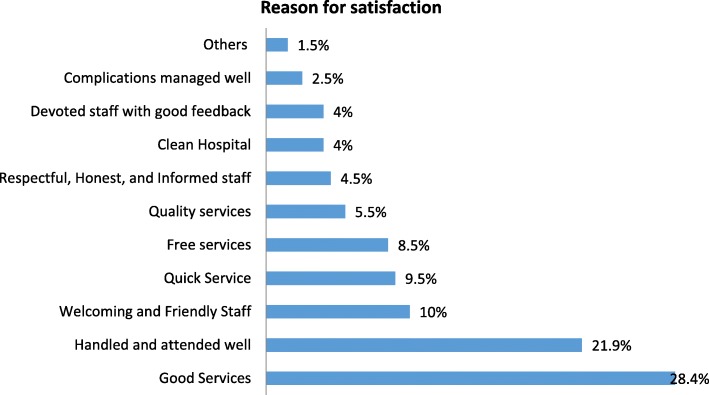
Reasons for satisfaction with the OBA services

**Fig. 2:**
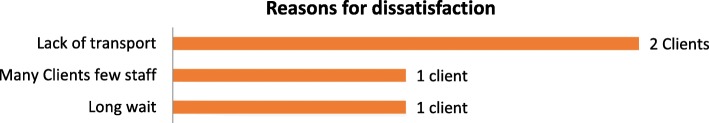
Reasons for dissatisfaction with the OBA services

All clients were asked whether they were completely satisfied with the services provided at the OBA sites. Ironically, 88.9% of the clients revealed they were satisfied despite the challenges with the issues that have been addressed above (Additional file 5: Figure S1). Satisfaction was presented using Pareto chart shown in Additional file 5: Figure S1 where reasons cited for satisfaction included courteousness by the staff and little waiting time to be seen by medical staff. Other reasons included welcoming and friendly staff (10%), free service (8.5%), and quality service (5.5%). On the other hand, two clients were dissatisfied with the service because of lack of transport to the facility while one client was dissatisfied because of long waiting time before being attended to by the staff (Figs. 1 and 2).

## Discussion

Our results show that F1- “staff conduct and practice” was judged relatively high. This shows that components of staff conduct and practice which are honesty, compassion, respect, openness, and devotion to work of healthcare workers provided a significant influence on the perceived quality of reproductive health services. Our findings are congruent with results from a study in Malawi which showed that women were overall satisfied with the level of maternal care at the facilities because they were respected, welcomed and listened to [31]. Our results also support the findings of a cross sectional study in Ghana of mothers who delivered vaginally in two public hospitals and revealed that they were treated with respect [32]. Additionally, the study is consistent with a study in Nicaragua where user satisfaction with vouchers was highly correlated to satisfaction with clinic reception and clarity of doctor’s explanations [33]. From the findings, we elucidate that women tend to associate the attitude of healthcare workers with the quality of care.

The quality of F2- “Healthcare delivery” was rated as relatively good. For instance, the respondents were happy with the competence of staff in the facilities who were capable of handling complications and giving enough information. This is analogous to a study in Malawi with respect to handling complications [34]. The findings were different from a study in Mulago, Uganda where only 38% of the mothers revealed that they had received adequate information on the symptoms and expected health problems [35]. However, in Serbia, mothers were content with the information given regarding their rights during and after delivery by the midwives which partly support our findings [34]. Additionally, women perceived that staff had adequate knowledge in dealing with SMH, LTFM, SGBV issues. These findings suggest that strong focus on the quality of care has contributed to increased service delivery in OBA sites.

Women judged F3 - “physical facilities”, F4 - “adequacy of resources”, and F5 - “Accessibility of care” as relatively moderate. Most women perceived that clean drinking water, availability of bathing facilities especially after delivery, and privacy when being examined, were essential components of a good healthcare facility. In essence, toilet and hand washing facilities enhanced the level of perceived quality of care. Moreover, within OBA sites, perceived quality of care was linked to adequate number of staff and the supply of drugs. Findings were comparable to a study in India, which indicated that women were happy with the readiness of primary drugs particularly during complications and availability of health workers [36]. Drugs are important determinants of quality of care and the absence of drugs could lead to impaired perception of the quality of services [10].

Our findings also reveal that women are content with majority of quality aspects despite the number of healthcare workers being low. This can probably be explained by the few number of health workers going way above their abilities and the workload to ensure that the mothers receive the services they need. Women seem to be aware of the shortage of workers, but appreciate the services they provide.

An important finding from this study was that the majority of respondents were young people of 24 years and below who made at least three ANC visits, which is comparable with the Kenya Demographic Health Survey (KDHS) 2014 results [9]. However, women needed relatively long hours to reach OBA facilities which was comparable to other studies [15, 19, 36] and greatly influenced women’ perception of the quality of care.

The study has revealed that area of residence played a key role in determining the level of perception of quality of care of OBA services as compared to other socio-demographic characteristics. However, the study identified some impact of ANC visit numbers, level of education, and age on the perception of quality which is in congruent with results from other sub-Saharan African studies [32, 37].

### Study limitations

In studies involving perception of quality and satisfaction with the level of care, there is a propensity to provide favorable answers to the questions [24]. Thus, in as much as the study is relevant, it should be used with caution. Besides, generalizing it to other countries is not warranted. Secondly, the sampling design provided enough users of OBA services to examine the research question; however, in some remotely located facilities, we did not find the designated number of women because they experienced difficulty in accessing the facilities. Thirdly, women were interviewed within the vicinity of the clinic or hospital and this may have influenced the way they answered the questions.

### Recommendations

- Health care managers can use our findings as a guide to evaluate different areas of healthcare delivery; thereby, improving resources and physical facilities that are crucial in elevating women’ level of satisfaction with the quality of care. Moreover, healthcare workers can use the study as a guide to enhance accessibility of care so that improved levels of satisfaction can be obtained.
- It is imperative for future programs to inculcate transport vouchers to reduce time to get to the facilities, as it is a potential determinant of perception of quality.
- For the program management unit (PMU), the index for perceived quality and women’ satisfaction should be incorporated into practice using the results from this study. While different facilities reacted differently to reimbursements and incentives, some facilities improved their structures and were able to attract more women who are more satisfied. Therefore, it is imperative to introduce mechanisms in the voucher strategies that can capture perceived quality and satisfaction routinely. The 23 item questions that have been translated into five factors shows the key areas that the PMU need to improve.

## Conclusion

Conduct and practice of healthcare workers is an important determinant of women’s perception of quality. Women take keen interest in evaluating staff attitudes. Healthcare workers within different areas of residence need to implement different strategies unique to the area that will pull and improve levels of satisfaction and perception of the quality of healthcare.

Women were overall satisfied with the way they were being handled at the OBA facilities. A future study could also assess whether healthcare providers’ perception of care is different from users’ perception. Policy makers should respect women’ quality perceptions within OBA services and work towards improving quality of care and enhancing utilization.

## Additional files

Additional file 1: Data collection tool for RH-OBA clients. (PDF 455 kb)

Additional file 2: **Table S1.** Reliability analysis of Factors and total score. (DOCX 13 kb)

Additional file 3: **Table S2.** Factors related to perceived quality: Multivariate response model for F1, F2, and F3. (DOCX 14 kb)

Additional file 4: **Table S3.** Factors related to perceived quality: Multivariate response model for F4, F5, and Total Score. (DOCX 17 kb)

Additional file 5: **Figure S1.** Pareto chart for the level of satisfaction of clients with the OBA services. (DOCX 17 kb)

## Abbreviations

FBOs: Faith Based Organisations
KDHS: Kenya Demographic Health Survey
LTFP: Long-Term Family Planning Methods
MDGs: Millennium Development Goals
NGOs: Non-Governmental Organisations
OBA: Output Based Approach
SGBV: Sexual Gender Based Violence
SMH: Safe Motherhood
