# Supplementary material for: A facility-based study of women’ satisfaction and perceived quality of reproductive and maternal health services in the Kenya output-based approach voucher program
Source: BMC Pregnancy Childbirth. 2018 Jul 28;18:310. doi: 10.1186/s12884-018-1940-9 (PMC6064123; doi:10.1186/s12884-018-1940-9)
Supplement: Supplementary file 1 — Data collection tool for RH-OBA clients. (PDF 455 kb) [file 12884_2018_1940_MOESM1_ESM.pdf]

**MINISTRY OF HEALTH**

# AN ASSESSMENT OF THE QUALITY OF RH-OBA SERVICES

# CLIENT-PERCEIVED QUALITY OF RH SERVICES IN SELECTED OBA SITES

## INTRODUCTIONS

- I am here today working on a project that will be assessing the quality of services in the OBA sites.
- We are collecting information from OBA clients who have benefitted from the OBA programme to understand their rating of the services they received.
- This information will be gathered by asking you some questions related to the service you received

**Purpose:** The information collected will be used to describe the level of satisfaction that clients have related to this service and thus inform on areas that require service improvement for effective and efficient RH-OBA implementation.

**Informed Consent (verbal):**

Are you willing to participate in a short interview with me of about 20 minutes, in which I will ask you about the maternity services you received. The questions asked will be about your thoughts and opinions on these issues. There are no right or wrong answers. Your responses will remain anonymous.

Your participation is completely voluntary. You may choose to stop answering at any point. Your participation, and the answers you provide, will not impact on your care at this hospital in any way.

**Date:** ___ / ___ / _______ **(DD-MM-YYYY)**

**County:** ______________________________________

**Sub-county:** ______________________________________

**Facility Name:** ______________________________________

**Enumerator's Name** ______________________________________

**Client Number:** ______________________________________

**SECTION A: GENERAL INFORMATION**

1. Age (years): ____________

2. Parity: _________+________

3. Education level (Tick one):

- ☐ No Education
- ☐ Primary Level
- ☐ Secondary Level
- ☐ Tertiary Level

4. Distance to reach facility from home (either by time on foot or by vehicle)

   4.1: time by foot: _____________ (Hrs/Minutes)

   4.2: time by vehicle: _____________ (Hrs/Minutes)

5. How many times have you attended the ANC clinic?

- ☐ Two or less No Education
- ☐ Three times or more

6. What is your marital status

- ☐ Never Married
- ☐ Married
- ☐ Not currently married

**SECTION B: PERCEIVED QUALITIES OF RH-OBA services**

Respondents will express their opinion or experience on a four-point Likert scale (1 - 5), where Completely Disagree (1), Disagree (2), Agree (3), Completely Agree (4) and Don't Know (5). For each aspect/question please tick in the appropriate box provided on the right.

| Aspects | Completely Disagree (1) | Disagree (2) | Agree (3) | Completely Agree (4) | Do not know (5) |
|---|---|---|---|---|---|
| **Q5: Health Facility** | | | | | |
| 5.1 In your opinion, the number of health staff in the health facility is **adequate.** | | | | | |
| 5.2 a) In your opinion, the health staff have **adequate** knowledge in dealing with FP, SGBV, CS, normal, deliveries. | | | | | |
| 5.2 b) In your opinion, the health staff in the health facility are **very capable** of finding out what is wrong with the patients. | | | | | |
| 5.2 c) You developed complications from using OBA services | | | | | |
| 5.2 d) In your opinion, complications arising from OBA services were dealt with effectively | | | | | |
| 5.3 a) In your opinion, the waiting rooms, examination rooms and other rooms of the health facility are **adequate** for RH-OBA services. | | | | | |
| 5.3 b) There is enough privacy while handling your cases | | | | | |
| 5.4 In your opinion, the provision of clean drinking water for OBA clients in the facility is **adequate.** | | | | | |
| 5.5 In your opinion, Hand washing facilities for OBA clients in the facility are **adequate.** | | | | | |
| 5.6 In your opinion, Bathing facilities for OBA clients in the facility are **adequate.** | | | | | |
| 5.7 In your opinion, Toilets for OBA clients in the facility are **adequate.** | | | | | |

| | | | | | |
|---|---|---|---|---|---|
| 5.8 In your opinion, the overall environment of the health facility is **very clean**. | | | | | |
| 5.9 In your opinion, the equipment in the health facility is **well suited** for detecting OBA client's medical problems and complications on time. | | | | | |
| 5.10) You received enough information on services you had come for to help you make informed decisions | | | | | |
| 5.11) In your opinion, the information of danger signs of delivery and postpartum provided by health staff is **adequate.** | | | | | |
| **Aspects** | **Completely Disagree (1)** | **Disagree (2)** | **Agree (3)** | **Completely Agree (4)** | **Do not know (5)** |
| **6. Health Care Delivery** | | | | | |
| 6.1 In your opinion, the health staff in the health facility examines pregnant and postpartum women **well**. | | | | | |
| 6.2 In your opinion, the health staff in the health facility prescribe the **drugs that are needed**. | | | | | |
| 6.3 In your opinion, there is adequate supply of drugs in this health facility. | | | | | |
| 6.4 In your opinion, patients can **easily** obtain drugs from this health facility. | | | | | |
| 6.6 You felt **very much** of unnecessary and humiliating procedures during antenatal and delivery care. | | | | | |
| | | | | | |

| **Aspects** | **Completely Disagree (1)** | **Disagree (2)** | **Agree (3)** | **Completely Agree (4)** | **Do not know (5)** |
|---|---|---|---|---|---|
| **7.0 Interpersonal Aspects** | | | | | |
| 7.1 In your opinion, the health staff in the health centre are **very open** with the patients. | | | | | |
| 7.2 In your opinion, the health staff in the health facility are **very compassionate** towards the patients. | | | | | |
| 7.3 In your opinion, the health staff are **respectful** towards the patients | | | | | |
| 7.4 In your opinion, the time that the health staff devote to their patients is **adequate**. | | | | | |
| 7.5 In your opinion, the health staff in the health facility are **honest.** | | | | | |
| **8.0 Overall** | | | | | |
| 8.1 You were **completely satisfied** with the services provided to you | | | | | |
| 8.2 In the case of your **future delivery** or next baby, will you again use this the health care facility? | yes | No | Undecided/do not know | | |
| 8.3 Is there any service you have paid for during your maternal care (ANC, Delivery, PNC, SGBV) | Yes | No | | If Yes, which service?<br>___________________<br>___________________ | |
