# Supplementary material for: A facility-based study of women’ satisfaction and perceived quality of reproductive and maternal health services in the Kenya output-based approach voucher program
Source: BMC Pregnancy Childbirth. 2018 Jul 28;18:310. doi: 10.1186/s12884-018-1940-9 (PMC6064123; doi:10.1186/s12884-018-1940-9)
Supplement: Supplementary file 2 — Table S1. Reliability analysis of Factors and total score. (DOCX 13 kb) [file 12884_2018_1940_MOESM2_ESM.docx]

Additional file 2: Table S1: Reliability analysis of Factors and total score

|  | Subscale | | | | | Perceived quality (Total score) |
| --- | --- | --- | --- | --- | --- | --- |
|  | F1 | F2 | F3 | F4 | F5 |  |
| **Number of items that converged** | 5 | 7 | 5 | 3 | 3 | 23 |
| **Possible Range** | -10 to +10 | -14 to +14 | -10 to +10 | -6 to +6 | -6 to +6 | -46 to +46 |
| **Mean** | 3.540 | 3.525 | 3.318 | 3.272 | 3.348 | 3.427 |
| **Median** | 4 | 4 | 3 | 3 | 3 | 3 |
| **Standard Deviations** | 0.587 | 0.609 | 0.708 | 0.818 | 0.975 | 0.807 |
| **Cronbach’s alpha** | 0.897 | 0.831 | 0.805 | 0.570 | 0.525 | 0.904 |
