# Supplementary material for: A facility-based study of women’ satisfaction and perceived quality of reproductive and maternal health services in the Kenya output-based approach voucher program
Source: BMC Pregnancy Childbirth. 2018 Jul 28;18:310. doi: 10.1186/s12884-018-1940-9 (PMC6064123; doi:10.1186/s12884-018-1940-9)
Supplement: Supplementary file 3 — Table S2. Factors related to perceived quality: Multivariate response model for F1, F2, and F3. (DOCX 14 kb) [file 12884_2018_1940_MOESM3_ESM.docx]

Additional file 3: Table S2: Factors related to perceived quality: Multivariate response model for F1, F2, and F3

| **Independent variables** | **Dependent variable (Scale)** | | | | | | | | | | |
| --- | --- | --- | --- | --- | --- | --- | --- | --- | --- | --- | --- |
|  | **Staff conduct and practice** | | |  | **Healthcare delivery** | | |  | **physical facilities** | | |
|  | **B^a^** | **95% CI** | |  | **B** | **95% CI** | |  | **B** | **95% CI** | |
|  |  | LB | UB |  |  | LB | UB |  |  | LB | UB |
| Intercept | **3.85^b^** | 3.761 | 3.934 |  | **3.531** | 3.460 | 3.603 |  | **3.693** | 3.583 | 3.802 |
| Level of Education (Ref: Primary Education) |  |  |  |  |  |  |  |  |  |  |  |
| No Education | 0.072 | -0.068 | 0.212 |  | -0.009 | -0.138 | 0.119 |  | **0.321** | 0.144 | 0.497 |
| Secondary Education | **0.082** | 0.010 | 0.154 |  | 0.058 | -0.008 | 0.124 |  | **0.104** | 0.013 | 0.195 |
| Tertiary Education | -0.042 | -0.150 | 0.066 |  | **-0.160** | -0.259 | -0.061 |  | -0.017 | -0.154 | 0.119 |
| ANC visits -Two or less | 0.030 | -0.042 | 0.103 |  | 0.054 | -0.013 | 0.120 |  | 0.091 | 0.000 | 0.183 |
| Marital Status (ref: Married) |  |  |  |  |  |  |  |  |  |  |  |
| Never Married | -0.019 | -.112 | .074 |  | -0.031 | -0.116 | 0.053 |  | -0.088 | -.205 | 0.029 |
| Separated/ Divorced | 0.128 | -.019 | .276 |  | -0.120 | -0.256 | 0.015 |  | -0.080 | -.267 | 0.107 |
| Age (Ref: 25-34) |  |  |  |  |  |  |  |  |  |  |  |
| 15-24 | **-0.084** | -0.149 | -0.019 |  | **-0.084** | -0.144 | -0.025 |  | -0.005 | -0.087 | 0.076 |
| 35-44 | 0.115 | -0.006 | 0.237 |  | 0.053 | -0.058 | 0.165 |  | 0.023 | -0.130 | 0.176 |
| County of residence (Ref: Kiambu) |  |  |  |  |  |  |  |  |  |  |  |
| Nairobi | **-0.745** | -0.879 | -0.612 |  | **-0.195** | -0.312 | -0.079 |  | **-0.685** | -0.852 | -0.518 |
| Kitui | **-0.373** | -0.481 | -0.265 |  | **-0.071** | -0.161 | 0.019 |  | **-0.528** | -0.664 | -0.391 |
| Kilifi | **-0.506** | -0.599 | -0.414 |  | -0.030 | -0.106 | 0.046 |  | **-0.568** | -0.684 | -0.451 |
| Kisumu | **-0.103** | -0.197 | -0.009 |  | -0.05 | -0.115 | 0.015 |  | **-0.406** | -0.525 | -0.286 |
| Variance Explained (R^2^) | 18.3% |  |  |  | 10.1% |  |  |  | 10.1% |  |  |
| 1. The B values shown are interpreted directly: for instance, 0.082 for secondary education on Factor 1 means that individual with secondary education give a score of 0.082 higher than the individual with primary education, after adjusting for other variables such as ANC visits, Marital status, Age, and county of residence 2. The bold values are significant at p < 0.05 | | | | | | | | | | | |
