# Supplementary material for: A facility-based study of women’ satisfaction and perceived quality of reproductive and maternal health services in the Kenya output-based approach voucher program
Source: BMC Pregnancy Childbirth. 2018 Jul 28;18:310. doi: 10.1186/s12884-018-1940-9 (PMC6064123; doi:10.1186/s12884-018-1940-9)
Supplement: Supplementary file 4 — Table S3. Factors related to perceived quality: Multivariate response model for F4, F5, and Total Score. (DOCX 17 kb) [file 12884_2018_1940_MOESM4_ESM.docx]

Additional file 4: Table S3: Factors related to perceived quality: Multivariate response model for F4, F5, and Total Score

| **Independent variable** | **Dependent variable (Scale)** | | | | | | | | | | |
| --- | --- | --- | --- | --- | --- | --- | --- | --- | --- | --- | --- |
|  | **Adequacy of resources** | | |  | **Accessibility of care** | | |  | **Perceived Quality (Total Score)** | | |
|  | **B^c^** | **95% CI** | |  | **B** | **95% CI** | |  | **B** | **95% CI** | |
|  |  | LB | UB |  |  | LB | UB |  |  | LB | UB |
| Intercept | **3.801** | 3.641 | 3.961 |  | **4.660** | 3.984 | 5.336 |  | **3.716** | 3.658 | 3.774 |
| Level of Education (Ref: Primary Education) |  |  |  |  |  |  |  |  |  |  |  |
| No Education | **0.365^d^** | 0.107 | 0.622 |  | 0.125 | -0.198 | 0.449 |  | **0.124** | 0.030 | 0.217 |
| Secondary Education | 0.100 | -0.033 | 0.232 |  | 0.073 | -0.092 | 0.239 |  | **0.101** | 0.053 | 0.149 |
| Tertiary Education | -0.102 | -0.301 | 0.097 |  | -0.043 | -0.291 | 0.206 |  | -0.052 | -0.125 | 0.020 |
| Two or less ANC visits | 0.022 | -0.111 | 0.156 |  | -0.049 | -0.216 | 0.118 |  | **0.053** | 0.004 | 0.101 |
| Marital Status (ref: Married) |  |  |  |  |  |  |  |  |  |  |  |
| Never Married | -0.010 | -0.180 | 0.161 |  | 0.001 | -0.212 | 0.213 |  | -0.029 | -0.091 | 0.033 |
| Separated/ Divorced | -0.170 | -0.442 | 0.102 |  | -0.087 | -0.431 | 0.256 |  | -0.036 | -0.135 | 0.063 |
| Age (Ref: 25-34) |  |  |  |  |  |  |  |  |  |  |  |
| 15-24 | 0.022 | -0.097 | 0.141 |  | **-0.860** | -1.505 | -0.215 |  | -0.042 | -0.086 | 0.001 |
| 35-44 | -0.068 | -0.291 | 0.156 |  | **-0.874** | -1.571 | -0.177 |  | 0.032 | -0.049 | 0.113 |
| County of residence (Ref: Kiambu) |  |  |  |  |  |  |  |  |  |  |  |
| Nairobi | **-0.753** | -0.996 | -0.510 |  | **-0.702** | -1.005 | -0.398 |  | **-0.620** | -0.709 | -0.532 |
| Kitui | **-1.023** | -1.222 | -0.825 |  | **-0.671** | -0.922 | -0.420 |  | **-0.482** | -0.554 | -0.410 |
| Kilifi | **-0.533** | -0.703 | -0.363 |  | **-0.575** | -0.788 | -0.362 |  | **-0.463** | -0.525 | -0.401 |
| Kisumu | **-0.600** | -0.774 | -0.427 |  | **-0.428** | -0.645 | -0.211 |  | **-0.351** | -0.415 | -0.288 |
| Variance Explained (R^2^) | 14.8% |  |  |  | 5.7% |  |  |  | 5.8% |  |  |
| 1. The B values shown are interpreted directly: for instance, 0.365 for no education on Factor 1 means that individual with no education give a score of 0.365 higher than the individual with primary education, after adjusting for other variables such as ANC visits, Marital status, Age, and county of residence 2. The bold values are significant at p < 0.05 | | | | | | | | | | | |
