# Supplementary material for: A facility-based study of women’ satisfaction and perceived quality of reproductive and maternal health services in the Kenya output-based approach voucher program
Source: BMC Pregnancy Childbirth. 2018 Jul 28;18:310. doi: 10.1186/s12884-018-1940-9 (PMC6064123; doi:10.1186/s12884-018-1940-9)
Supplement: Supplementary file 5 [file 12884_2018_1940_MOESM5_ESM.docx]

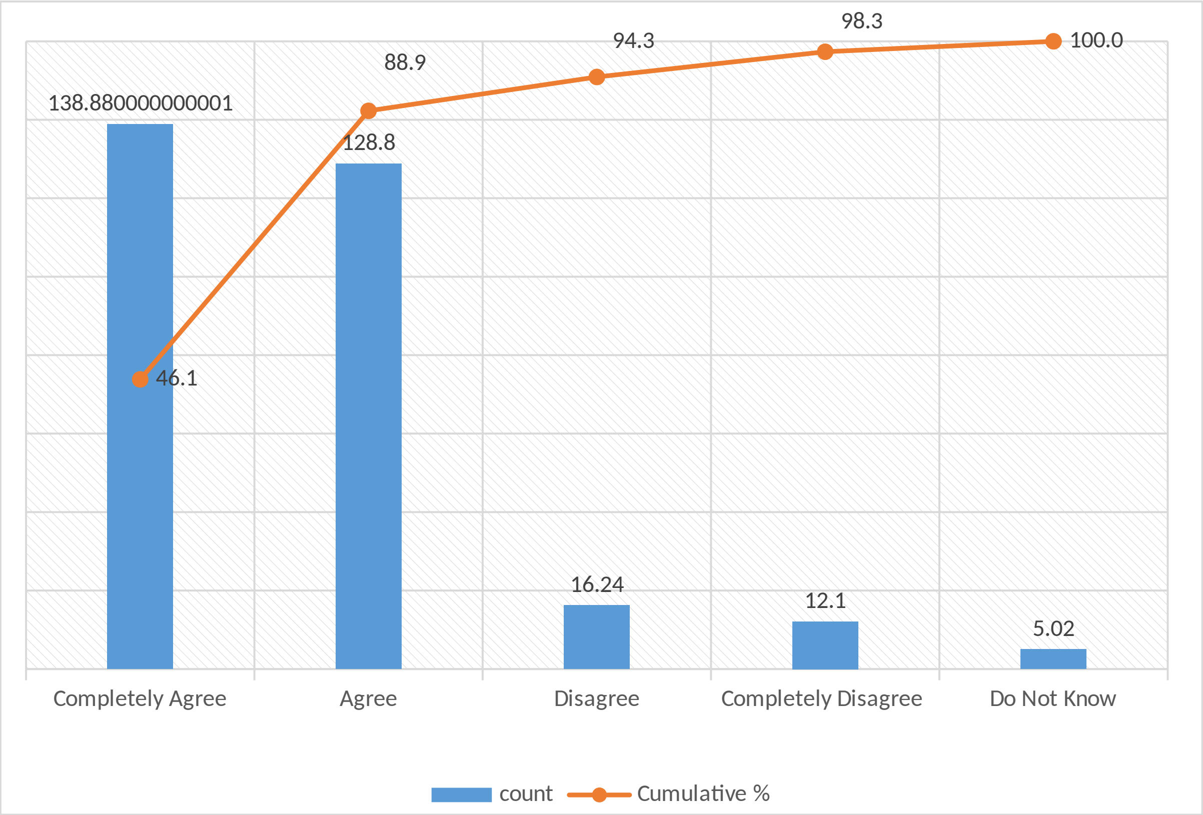

138.880000000001
128.8
16.24
12.1
5.02
46.1
88.9
94.3
98.3
100.0
Completely Agree
Agree
Disagree
Completely Disagree
Do Not Know
count
Cumulative %
